# Case Report: Four Early Recurrent Basilar Artery Occlusions Successfully Treated With Mechanical Thrombectomy and Subsequent Vertebral Artery Coil Occlusion

**DOI:** 10.3389/fneur.2021.698488

**Published:** 2021-09-20

**Authors:** Stefan L. Leber, Simon Fandler-Höfler, Markus Kneihsl, Michael Augustin, Hannes Deutschmann, Thomas Gattringer

**Affiliations:** ^1^Division of Neuroradiology, Vascular and Interventional Radiology, Department of Radiology, Medical University of Graz, Graz, Austria; ^2^Department of Neurology, Medical University of Graz, Graz, Austria

**Keywords:** thrombectomy, stroke, recurrent stroke, large vessel occlusion, basilar artery occlusion, endovascular treatment (EVT), ischemic stroke

## Abstract

We present the case of a middle-aged patient who had four recurrent acute basilar artery occlusions over a period of 3 months, each time successfully treated with mechanical thrombectomy. Extensive stroke work-up showed no obvious stroke etiology aside from a dysplastic right vertebral artery with multifocal stenoses. Treatment with different antiplatelet and anticoagulant regimes did not prevent basilar artery occlusion recurrence. Therefore, transarterial coil occlusion of the V4-segment of the right vertebral artery was performed as ultima ratio without complications. At final discharge, the patient had no persistent neurological deficits. No further cerebrovascular events occurred over a 12-month follow-up period.

## Introduction

Basilar artery occlusion (BAO) entails a high risk for severe disability and mortality. The role of mechanical thrombectomy (MT) in large vessel obstruction of the posterior cerebral circulation has been discussed controversially (1). However, more recent data support an effective role of MT in BAO (2, 3). Especially data on treatment strategies of recurrent BAO are scarce, with only few cases published (4–8). A case of four recurrent BAOs refractory to various medical treatment regimens has not yet been described in the literature.

## Case Description

A 53-year-old man presented with acute onset of dizziness, left-sided hemiparesis and progressive loss of consciousness. CT angiography (CTA) revealed BAO and additionally showed a calcified dysplastic right vertebral artery (VA) with multifocal stenoses. One month earlier, the patient had suffered from a bithalamic infarct treated with intravenous thrombolysis (IVT). The patient recovered well and had been discharged with atorvastatin and dual antiplatelet therapy with aspirin and clopidogrel, though aspirin had been discontinued few days before readmission. Vascular risk factors included dyslipidemia, coronary heart disease and peripheral artery disease.

**Figure 1:**
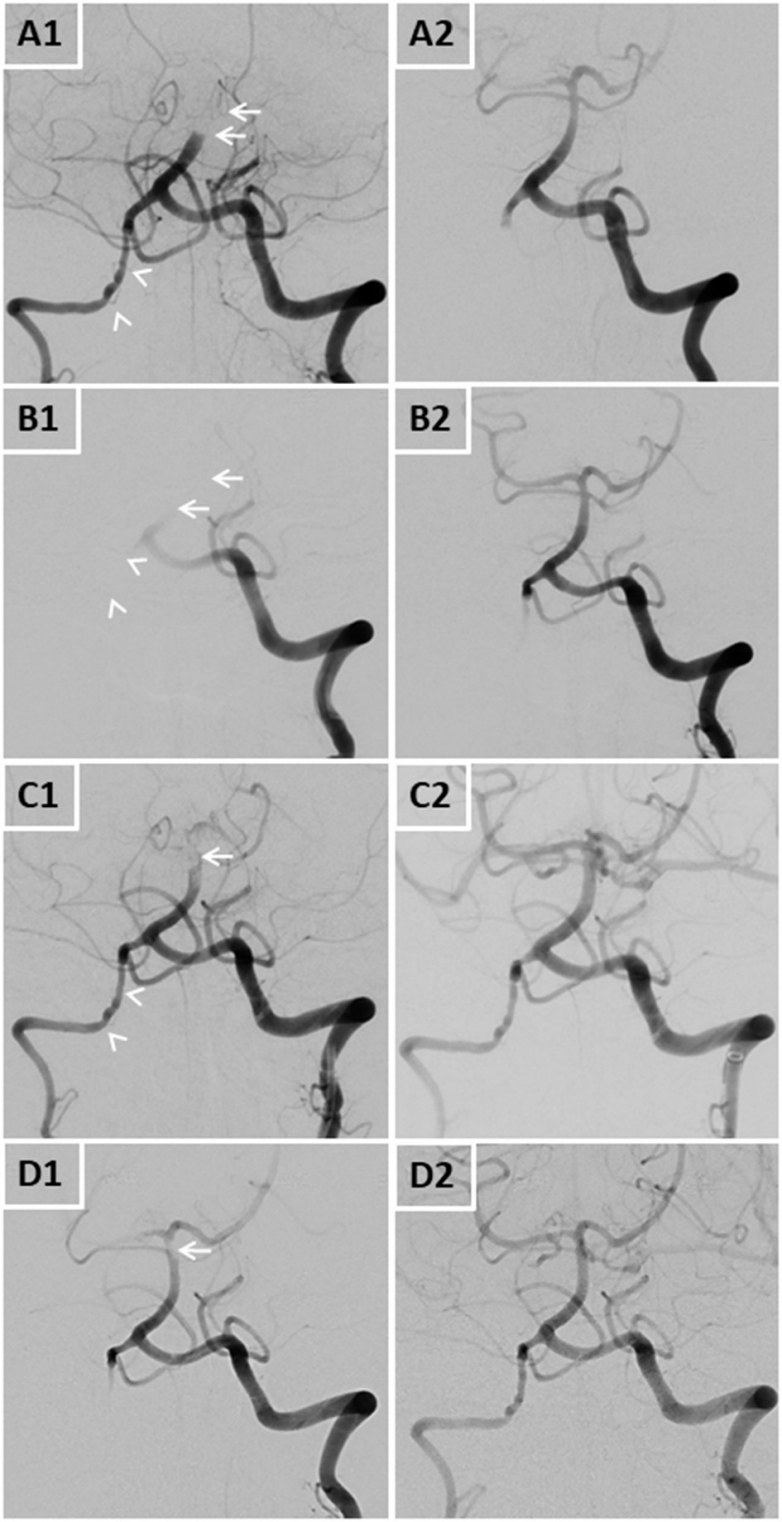
DSA images of all four basilar artery occlusions pre- **(A1,B1,C1,D1)** and post-interventionally **(A2,B2,C2,D2)**. Arrows display arterial occlusions and/or thrombi, arrowheads display the dysplastic right vertebral artery.

IVT and MT achieved complete recanalization (Figures 1A1,A2) and an excellent neurological outcome with a modified Rankin Scale (mRS) of 0 and a National Institutes of Health Stroke Scale (NIHSS) of 0. Aspirin and subcutaneous enoxaparin were initiated during stroke work-up and early rehabilitation. Nine days post-thrombectomy, while still in hospital, the patient suddenly developed acute left-sided hemiparesis and dysarthria. MR angiography (MRA) showed distal BAO; subsequently direct MT was performed. Again, full recanalization was achieved and the patient remarkably improved (mRS 0/NIHSS 0) (Figures 1B1,B2). Two weeks later, the patient was discharged without any neurological deficits with a combination therapy of dabigatran, aspirin and atorvastatin due to the recurrent BAO. Notably, he did not take any additional drugs potentially interfering with blood clotting (such as nonsteroidal anti-inflammatory drugs).

Four days after discharge, the patient was readmitted with left-sided hemiparesis and dysarthria. CTA confirmed the third recurrent BAO, and the patient was again successfully recanalized with MT (Figures 1C1,C2). Follow-up MRI displayed new small infarcts in both occipital lobes and both cerebellar hemispheres, but again the patient had very good clinical outcome (mRS 0/NIHSS 0). Platelet aggregation testing showed a very good response to aspirin. After further stroke work-up and early rehabilitation, the patient was discharged with no persisting deficits and switched to a combination treatment of phenprocoumon and aspirin.

One week after discharge, the patient again presented with vertigo and left-sided sensory deficits. MRA again showed a thrombus in the distal basilar artery and the proximal left posterior cerebral artery, which were removed by endovascular therapy using an aspiration catheter (Figures 1D1,D2).

Extensive stroke work-up was performed. Transesophageal echocardiography excluded relevant structural cardiac abnormalities, ECG monitoring at the neurocritical care and stroke unit as well as repeated 24 h ECG did not reveal paroxysmal atrial fibrillation. Dyslipidemia was well-controlled with atorvastatin. Extensive laboratory tests, full-body PET and MRA, lumbar puncture and flow cytometry analysis excluded vasculitis, collagenosis, cancer and antiphospholipid antibody syndrome. However, MRA and CTA repeatedly showed a dysplastic right vertebral artery with multifocal stenoses. The family history of the patient was unremarkable.

After ruling out numerous etiologies for recurrent BAO and as all strokes occurred in the same arterial territory, arterio-arterial embolism originating from the dysplastic multifocally stenosed right VA was regarded as the presumed cause, with the most likely underlying process being either atherosclerosis or fibromuscular dysplasia (FMD).

**Figure 2:**
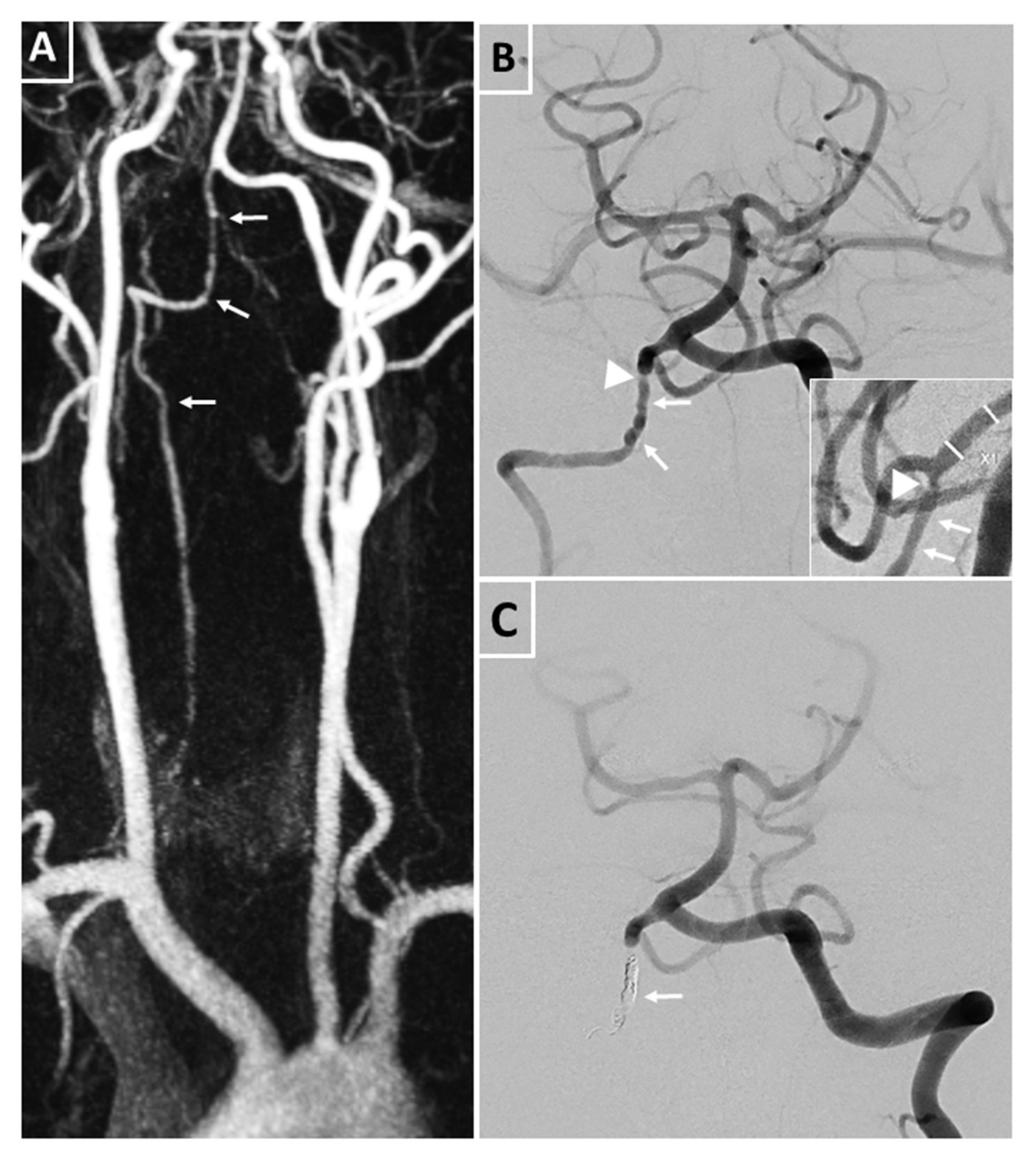
Pre- and post-interventional images of the right dysplastic vertebral artery and its multifocal stenoses. **(A)** MRA displaying the right vertebral artery with multiple intra- and extracranial irregularities (tailed arrows) corresponding to the diagnosis of a dysplastic vertebral artery. **(B)** Pre-interventional digital subtraction angiography of the posterior circulation with irregularities (tailed arrows) and distal stenosis of the vertebral artery (arrow head) proximal to the outflow of the right posterior inferior cerebellar artery in two plains. **(C)** Post-interventional digital subtraction angiography of the posterior circulation after coil occlusion of the right V4 segment (tailed arrow) with preservation of the right posterior inferior cerebellar artery outflow.

As different aggressive antithrombotic medical treatments had failed to prevent recurrent BAO, we decided for a deconstructive interventional approach after careful consideration and elaborate discussion with the patient. Since blood supply by the left VA seemed sufficient for the posterior cerebral circulation, transarterial coil-occlusion of the right VA was performed. The proximal V4-segment was occluded in a cross-over technique with multiple platinum coils, whereas the right PICA and the distal V4-segment were preserved (Figures 2A–C).

A 6F sheath (Neuron Max 0.88, Penumbra, Inc., CA, USA) was placed in the left vertebral artery. Dual microcatheter technique was applied to exactly place the platinum coils. The first microcatheter (Excelsior SL10, Stryker, CA, USA) was exactly positioned proximal to the origin of the PICA, and a coil (Smart coil, Penumbra, Inc., CA, USA) was placed but not released. The second microcatheter (Excelsior SL10, Stryker, CA, USA) was placed proximal to the first, at the beginning of the V4 segment. Through this microcatheter, seven platinum coils were placed. Only after confirmation of the correct and safe positioning of the coils without compromising the origin of the PICA, the first coil was released.

**Figure 3:**
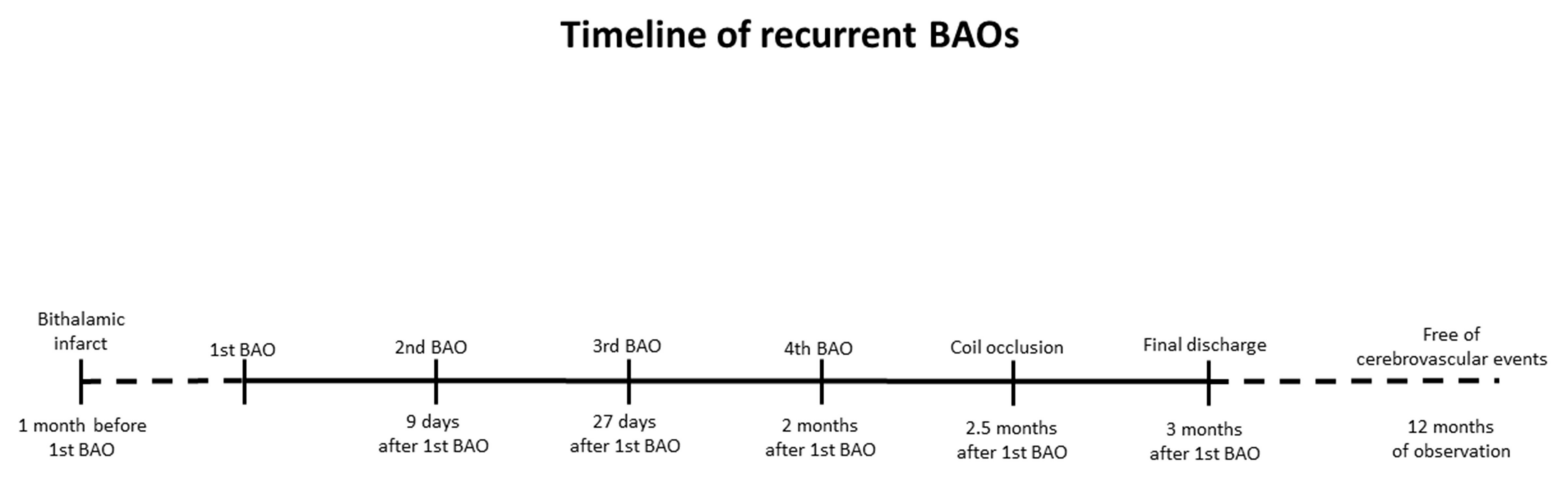
Timeline of stroke events.

The patient was ultimately discharged without focal neurological deficits/residual symptoms (mRS 0/NIHSS 0) and with dual antiplatelet therapy (aspirin and clopidogrel) in order to minimize risk of recurrent atherothrombotic events. Clopidogrel was discontinued after 6 months. No recurrent cerebrovascular events occurred during a 12-month follow-up period. Figure 3 depicts a full timeline of the mentioned events.

## Discussion

We present a patient with four recurrent BAO that had occurred during a remarkably short time period and were all treated successfully with endovascular stroke therapy leading to excellent outcome. Notably, a multifocally stenosed right VA was identified as the most likely underlying cause. This apparently highly aggressive arterial embolic source could not be controlled with different medical strategies. Therefore, successful coil occlusion of the V4 segment of the VA was performed. During the follow-up period, no further cerebrovascular events occurred.

Recurrent BAO was reported in the context of VA dissection (3), neoplasia (5), antiphospholipid syndrome (6) and VA stenosis (5). A recent case series on recurrent cerebral large vessel occlusion suggested that periinterventional endothelial lesions might contribute to recurrent large vessel occlusions (4). Notably, we did not detect such lesions in any angiographic series and no other intervention-associated complications such as dissections or vasospasms had occurred in our patient.

Therapeutic VA occlusion has been described in the context of ruptured dissecting aneurysms, external VA compression or pre-operative intervention in cervical spine trauma (9–11). Our case is the first report of recurrent BAO with VA occlusion as final therapy. Successful recanalization by MT in every instance, good collateralization, a short symptom to recanalization time as well as site and size of thrombi contributed to the favorable outcome of our patient (3).

Regarding the stenosed right VA, our patient's history of coronary heart and peripheral artery disease suggest an atherosclerotic cause as source of recurrent embolism. However, the inefficacy of aggressive antiplatelet and anticoagulation therapy points toward an alternative non-atherosclerotic mechanism, such as focal FMD.

## Data Availability Statement

The original contributions presented in the study are included in the article, further inquiries can be directed to the corresponding author.

## Ethics Statement

Ethical review and approval was not required for the study on human participants in accordance with the local legislation and institutional requirements. The patients/participants provided their written informed consent to participate in this study. Written informed consent was obtained from the individual(s) for the publication of any potentially identifiable images or data included in this article.

## Author Contributions

SL, SF-H, and TG were involved in the conception of this case report. SL wrote the manuscript draft. SF-H supervised the study. All authors were involved in the treatment of the patient and revised the manuscript for important intellectual content, read, and approved the submitted version.

## Conflict of Interest

The authors declare that the research was conducted in the absence of any commercial or financial relationships that could be construed as a potential conflict of interest.

## Publisher's Note

All claims expressed in this article are solely those of the authors and do not necessarily represent those of their affiliated organizations, or those of the publisher, the editors and the reviewers. Any product that may be evaluated in this article, or claim that may be made by its manufacturer, is not guaranteed or endorsed by the publisher.
